# Estimation of Citrus Leaf Relative Water Content Using CWT Combined with Chlorophyll-Sensitive Bands

**DOI:** 10.3390/s26020467

**Published:** 2026-01-10

**Authors:** Xiangqian Qi, Yanfang Li, Shiqing Dou, Wei Li, Yanqing Yang, Mingchao Wei

**Affiliations:** 1 School of Resource Engineering, Longyan University, Longyan 364012, China; liyanfang@lyun.edu.cn; 2 College of Geomatics and Geoinformation, Guilin University of Technology, Guilin 541006, China; doushiqing@glut.edu.cn; 3 Department of Mining Engineering, Heilongjiang University of Science and Technology, Harbin 150022, China; iamink@163.com (W.L.); yangyanqing@usth.edu.cn (Y.Y.); weimingchao@usth.edu.cn (M.W.)

**Keywords:** citrus leaf relative water content, LCC, CWT, SPA

## Abstract

In citrus cultivation practice, regular monitoring of leaf leaf relative water content (RWC) can effectively guide water management, thereby improving fruit quality and yield. When applying hyperspectral technology to citrus leaf moisture monitoring, the precise quantification of RWC still needs to address issues such as data noise and algorithm adaptability. The noise interference and spectral aliasing in RWC sensitive bands lead to a decrease in the accuracy of moisture inversion in hyperspectral data, and the combined sensitive bands of chlorophyll (LCC) in citrus leaves can affect its estimation accuracy. In order to explore the optimal prediction model for RWC of citrus leaves and accurately control irrigation to improve citrus quality and yield, this study is based on 401–2400 nm spectral data and extracts noise robust features through continuous wavelet transform (CWT) multi-scale decomposition. A high-precision estimation model for citrus leaf RWC is established, and the potential of CWT in RWC quantitative inversion is systematically evaluated. This study is based on the multi-scale analysis characteristics of CWT to probe the time–frequency characteristic patterns associated with RWC and LCC in citrus leaf spectra. Pearson correlation analysis is used to evaluate the effectiveness of features at different decomposition scales, and the successive projections algorithm (SPA) is further used to eliminate band collinearity and extract the optimal sensitive band combination. Finally, based on the selected RWC and LCC-sensitive bands, a high-precision predictive model for citrus leaf RWC was established using partial least squares regression (PLSR). The results revealed that (1) CWT preprocessing markedly boosts the estimation accuracy of RWC and LCC relative to the original spectrum (max improvements: 6% and 3%), proving it enhances spectral sensitivity to these two indices in citrus leaves. (2) Combining CWT and SPA, the resulting predictive model showed higher inversion accuracy than the original spectra. (3) Integrating RWC Scale7 and LCC Scale5-2224/2308 features, the CWT-SPA fusion model showed optimal predictive performance (R^2^ = 0.756, RMSE = 0.0214), confirming the value of multi-scale feature joint modeling. Overall, CWT-SPA coupled with LCC spectral traits can boost the spectral response signal of citrus leaf RWC, enhancing its prediction capability and stability.

## 1. Introduction

Citrus products have become one of the core categories in both domestic and international agricultural markets due to their stable market demand and high economic returns. China accounts for 73.06% of the world’s citrus planting area and 84.12% of the world’s output. In 2023, the citrus planting area in Guangxi will reach 9.59 million Mu, with a yield of 18.95 million tons. It is the core production area of China’s citrus industry and has an important impact on the national supply chain. The leaf water status directly regulates the key physiological processes of plants and is the basis for maintaining the normal metabolism of plants [1], and as a medium and participant in biochemical reactions, the water status directly determines the light energy conversion efficiency and mineral nutrient allocation dynamics of plants [2]. Chlorophyll, as one of the main pigments involved in photosynthesis, not only affects the growth and yield of citrus plants [3] but also provides important indicators for monitoring crop-related growth information [4]. It interacts with water content indicators and has a certain correlation. Therefore, further exploration of chlorophyll information during the growth process of citrus plants has a very important guiding role in assisting in monitoring the moisture content of citrus leaves.

In recent years, an increasing amount of research has used hyperspectral technology to monitor plant growth indicators such as chlorophyll [3], nitrogen content [5], and leaf water content [6], all of which have achieved good results. At present, most studies use hyperspectral data and related spectral preprocessing to invert a single indicator of plants. There are few studies that have jointly explored and analyzed the correlations between multiple plant indicators and their impacts on inversion, such as the correlations between moisture content and chlorophyll information among plant physiological indicators. Under certain water stresses, the chlorophyll content is strongly affected [7]. With increasing water stress, the soil and plant analysis development (SPAD) value of the relative chlorophyll content gradually decreases [8]. Water stress can simultaneously affect changes in relative water content and photosynthetic pigments [9]. Therefore, the water content of leaves and leaf chlorophyll have a mutual influence under certain conditions. Currently, there is no report in the academic community on using continuous wavelet transform (CWT) technology to simultaneously probe the sensitive characteristic bands of relative water content (RWC) and LCC in citrus leaves, especially the effect of CWT enhanced chlorophyll spectral features on improving the accuracy of RWC inversion still needs systematic research.

High-precision hyperspectral data is predominantly obtained using portable ASD spectrometers in field-based measurements. However, due to the inherent electronic noise interference of sensor systems and random errors originating from the internal signal conversion process of spectrometers [10], the overlap of noise and effective information in the wavelength band [11], and the influence of exceptional states such as the viscosity and bit size of substances and experimental conditions on the spectral curve [12], the effective information of the spectrum is affected. Therefore, it is very important to make reasonable use of spectral preprocessing methods. Common spectral preprocessing methods include SG, SD, MSC, and combinations of preprocessing methods [13]. Although traditional preprocessing methods can improve signal-to-noise ratio, their effectiveness in unmixing spectral peaks in overlapping bands is limited. The CWT, with its rich wavelet basis functions and multi-resolution analysis advantages, can decompose leaf reflectance spectra into many scale components. Scholars have quickly determined the model inversion accuracy of cotton crop moisture content, nitrogen content, and SPAD value using this method [14], citrus leaf chlorophyll content [3], and winter wheat canopy leaf moisture content [15]. At present, there is no systematic application of CWT technology to probe the sensitive spectral characteristics of citrus leaf RWC and LCC, especially the effect of CWT enhanced chlorophyll spectral characteristics on improving RWC inversion accuracy still needs further exploration.

Hyperspectral data, while rich in spectral information across thousands of bands, inevitably contain substantial noise and irrelevant variables [16]. The critical challenge lies in effectively identifying and extracting the most informative spectral features for accurate citrus leaf water content estimation. The Successive Projections Algorithm (SPA) has emerged as a powerful tool for spectral feature optimization [17]. It can screen feature bands by minimizing collinearity effects in the calibration dataset, thereby eliminating redundant data and improving model efficiency [18].

In order to explore the optimal prediction model for RWC of citrus leaves and accurately control irrigation to improve citrus quality and yield, this study takes the Seedless Wogan Practice Base in Yanshan District, Guilin City, as the research area (25°16′48′′ N, 110°20′24′′ E). Collect RWC, LCC, and in situ spectral data of citrus leaves using relevant instruments and equipment, and decompose the in situ spectra into multiple wavelet scales through CWT. Using SPA to screen the characteristic sensitive bands of RWC and LCC in citrus leaves, and then using partial least squares regression (PLSR) to fuse the optimal band combination of two parameters, a multi physiological parameter collaborative citrus leaf moisture predictive model is constructed. The research objectives of this paper are as follows:

(1) This study verifies whether the multi-scale decomposition using CWT can improve the prediction accuracy of RWC and LCC and evaluates the effectiveness of this method;

(2) This study uses SPA to screen the sensitive bands of RWC and LCC, and analyzes their distribution characteristics, in order to explore the optimal combination of sensitive bands to improve the accuracy of RWC prediction models;

(3) This study aims to identify the optimal prediction model for RWC in citrus leaves by analyzing the sensitive band combinations of RWC and LCC, providing assistance for precision agriculture.

## 2. Materials and Methods

### 2.1. General Situation

**Figure 1:**
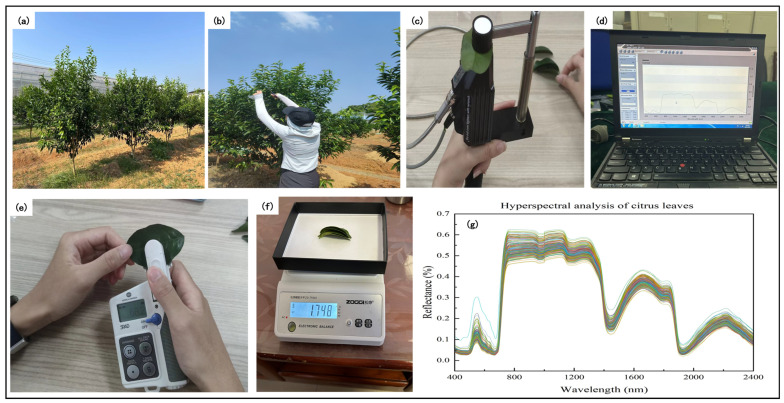
General situation. (**a**) Growth status of fruit trees; (**b**) leaf collection location; (**c**) measurement spectrum; (**d**) original spectral curve; (**e**) measure SPAD; (**f**) measure weight; and (**g**) 401–2400 nm leaf reflectance.

Guilin, as the largest citrus planting area in Guangxi, is renowned for its year-round supply of high-quality citrus fruits. Guilin has typical subtropical monsoon climate characteristics, with an annual average temperature of 18.5–19.5 °C, an annual precipitation of 1500 mm, sufficient sunshine, and a water heat coefficient that is highly consistent with the water demand pattern of citrus. Cooperating with the deep soil layer developed from limestone parent material, the organic matter content is abundant, forming an ideal habitat for citrus golden planting belt. The cultivation conditions are good, which can keep citrus fruit trees in the best growth state. This study takes the Seedless Wogan Practice Base in Yanshan District, Guilin City, as the research area, and the research object is citrus fruit trees planted in the study area for about three years. The spacing between planting rows is 3 m, and the spacing between citrus fruit trees is 2 meters as shown in Figure 1a. The citrus leaf collection is shown in Figure 1b.

### 2.2. Data and Spectral Collection

#### 2.2.1. Sample Collection

The experimental data were collected on the morning of 30 October 2023, in the research area of the Seedless Wogan Practice Base. A total of 58 sample fruit trees were selected, and 5 healthy and intact leaves were collected from the upper part of each fruit tree in four directions: north, south, east, and west. After collection, they were immediately sealed in sealed bags and stored in insulated boxes to prepare for subsequent measurement of leaf growth indicators. Finally, a total of 232 samples of fruit trees were collected. For the 232 collected leaf samples, first measure the fresh weight of each leaf sample, then measure the SPAD of each leaf, measure the original spectrum of each leaf, and finally measure the dry weight of each leaf to calculate the RWC.

#### 2.2.2. Spectral Acquisition

This study used ASD (FieldSpec 4 Stand Res, Boulder, CO, USA) to obtain original hyperspectral data of 232 samples of fruit tree leaves. Before using ASD to obtain original spectra in the laboratory, the instrument needs to be preheated for at least 15 min and kept at room temperature stable. After preheating, use a calibration whiteboard for calibration. When measuring the spectrum of the blade, the main pulse of the blade should be avoided from the light path and kept opaque, as shown in Figure 1c. After ensuring the stability of the spectral curves, 10 stable spectral curves were saved under the condition of taking an average spectrum for every 10 spectra, as shown in Figure 1d, to obtain the in situ spectral data of the sample fruit tree leaves. In the process of obtaining spectra, it is necessary to recalibrate the instrument based on the stability of the spectral curve to ensure the accuracy of the spectral data. Due to the decrease in quantum efficiency of the detector, the spectral signal-to-noise ratio significantly deteriorates in the near ultraviolet region (350–400 nm) and near-infrared region (2401–2500 nm), with a decrease of 4–6 times compared to the normal band [19]. Therefore, the spectral reflectance in these two ranges was excluded, as shown in Figure 1g.

#### 2.2.3. Chlorophyll Measurement

There are two main methods for determining the chlorophyll content of leaf samples collected in the field: direct laboratory chemical analysis using spectrophotometry and indirect determination using a portable chlorophyll instrument SPAD-502. The spectrophotometric method is based on the characteristic absorption peaks of chlorophyll at 645 nm and 663 nm, while SPAD-502 establishes an empirical relationship with chlorophyll content by measuring the ratio of transmitted light intensity at 650 nm and 940 nm [20]. Studies have shown that SPAD values have a significant power function relationship with LCC, and the correlation is strong ($R^{2}$ > 0.9023) [21]. Therefore, this value is considered the relative chlorophyll content and is widely used in ecological agriculture surveys and vegetation remote sensing fields.

In this study, a SPAD-502 (Konika-Minolta, Japan) handheld chlorophyll instrument was used to measure the SPAD value of citrus as the citrus leaf LCC. Using non-invasive measurement methods, the tested blade is placed in the measurement light path, and the probe is lightly closed until the trigger pressure is reached to automatically complete the detection without damaging the blade tissue structure throughout the entire process, as shown in Figure 1e.

#### 2.2.4. Chlorophyll Measurement Actual Measurement of Water Content

This study used an oven drying method to determine the RWC of citrus leaves, and electronic balance (ZG-TP203, China) was used for weight measurement, as shown in Figure 1f. Place the collected fruit tree leaves in a 105 °C blast drying oven for initial drying for 30 min, then continue drying at 80 °C. After the dryer cools down, use an analytical balance with an accuracy of 0.1 mg to measure the fresh weight (FW) and dry weight (DW) of citrus leaves. Calculate RWC using FW and DW as shown in Equation (1):

(1) $$RWC=\frac{FW-DW}{FW}\times 100\%,$$

Divide the sample into a training set (185) and a testing set (47) in a 4:1 ratio.

### 2.3. Calculation Methods

This study is based on the broadband spectrum hyperspectral data of citrus leaves. Innovatively establish a CWT-SPA two-stage feature selection framework, extract spectral features through CWT multi-scale decomposition, apply SPA to screen sensitive bands of RWC and LCC, analyze the distribution of sensitive bands, and construct a PLSR model to evaluate the impact of band combinations on inversion accuracy, and finally combine the sensitive bands of RWC and LCC in citrus leaves to explore the optimal predictive model of RWC in citrus leaves.

#### 2.3.1. Continuous Wavelet Transform

CWT achieves multi-resolution analysis of signals in the time–frequency domain by scaling and shifting the mother wavelet function, ensuring lossless reconstruction of information. CWT has adaptive resolution, high temporal resolution in the high-frequency region, high frequency resolution in the low-frequency region, and can ensure no missing features, making it particularly suitable for processing non-stationary spectral signals [22].

In CWT applications, the choice of wavelet function significantly influences spectral processing outcomes. After comparing and analyzing multiple wavelets including Morlet, Mexican Hat, Cmor, Cgau, and Fbsp, the Gaus family offers distinct advantages for spectral analysis: its minimal oscillation and smooth transition characteristics ensure stable signal processing while effectively preserving local spectral features. Particularly for analyzing transient and localized spectral characteristics, Gaussian wavelets demonstrate superior performance compared to other wavelet functions. For these reasons, this study employs the Gaus1 to decompose citrus leaf spectra. This selection optimally balances feature preservation and noise reduction, making it particularly suitable for detecting subtle spectral variations related to leaf water content.

The mathematical meaning of CWT is expressed in formula as shown in Equation (2):

(2) $$W\left(a,b\right)=\frac{1}{\surd a}\int_{-\infty }^{+\infty }x\left(t\right)\Psi^{*}\left(\frac{t-b}{a}\right)dt,$$

where x(t) is the input signal, $\Psi^{*}(t)$ is the conjugate of the wavelet function, a and b represent the scale and shift, respectively, and W(a,b) is the corresponding wavelet coefficient. The CWT is used to map one-dimensional spectral reflectance signals into two-dimensional time–frequency matrices, the formulas are shown in Equations (3) and (4):

(3) $$W_{f\left(a,b\right)}=\int_{-\infty }^{+\infty }f(\lambda )\psi_{a,b}(\lambda )d\lambda ,$$

(4) $$\Psi_{a,b}\left(\lambda \right)=\frac{1}{\sqrt{a}}\Psi \left(\frac{\lambda -b}{a}\right),$$

where $W_{f\left(a,b\right)}$ is the wavelet coefficient, f(λ) is the leaf spectral reflectance, λ is the spectral band in the range of 401~2400 nm, and $\Psi_{a,b}\left(\lambda \right)$ is the Gaussian wavelet function transformed by scale factor a and scaling factor b.

The CWT improves prediction accuracy by decomposing in situ spectra into multiple scales under certain conditions [23]. This study used the Gaus1 wavelet basis function to decompose the 401–2400 nm citrus leaf reflectance spectra into 10 logarithmic distribution scales. By systematically evaluating the correlation between wavelet coefficients at each scale and leaf RWC, the optimal feature extraction scale was determined.

#### 2.3.2. Successive Projections Algorithm

SPA is an effective feature selection method designed to enhance the performance of multivariate regression models by reducing multicollinearity in calibration datasets [24]. By identifying and retaining only the most informative wavelengths from spectral data, SPA efficiently eliminates redundant variables while preserving critical spectral features. Due to its ability to minimize data dimensionality without sacrificing predictive power, this algorithm has become a widely adopted tool for extracting optimal characteristic wavelengths in spectral analysis.

### 2.4. Regression Model and Accuracy Evaluation

This study adopts the CWT-SPA-PLSR technology route, based on Gaus1 wavelet for 10 scale decomposition, applies SPA to screen the sensitive bands of citrus RWC and LCC, explores the correlation between the two-growth index sensitive bands, and then constructs a PLSR model that integrates RWC–LCC-sensitive bands to explore the optimal predictive model for citrus leaf RWC.

**Figure 2:**
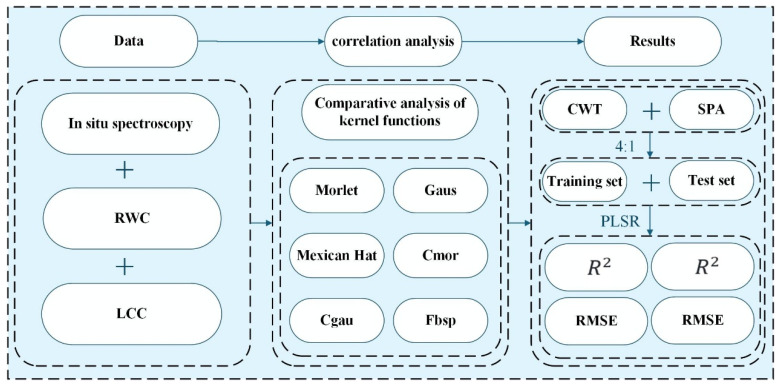
Experimental flowchart.

This study evaluated the accuracy of the inversion results of citrus leaf water content using the coefficient of determination ($R^{2}$), root mean square error (RMSE), and residual prediction deviation (RPD). The model optimization follows the following criteria, with $R^{2}$ approaching 1 and RMSE approaching 0. The higher the $R^{2}$, the smaller the RMSE, and the better the model accuracy. RPD is a critical metric for assessing the predictive capacity of a model. RPD between 1.4 and 2.5 suggests acceptable predictive ability and RPD exceeding 2.5 signifies strong model stability and accuracy and is suitable for practical application [25]. The equations are shown in (5)–(7):

(5) $$R^{2}=1-\frac{\sum_{i=1}^{n}{\left(y_{i}-\hat{y_{i}}\right)}^{2}}{\sum_{i=1}^{n}{\left(y_{i}-\bar{y_{i}}\right)}^{2}},$$

(6) $$RMSE=\sqrt{\frac{{\sum_{i=1}^{n}\left(\hat{y_{i}}-y_{i}\right)}^{2}}{n}},$$

(7) $$RPD=\frac{1}{\sqrt{1-R^{2}}},$$

where n is the number of samples, $y_{i}$ is the measured value of the citrus leaf water content, $\hat{y_{i}}$ is the predicted value of the citrus leaf water content, and $\bar{y_{i}}$ is the average value of the leaf water content. Below is the flowchart of our entire experiment, as shown in Figure 2.

## 3. Results

### 3.1. Correlation Analysis Between RWC and LCC Based on PLSR

**Figure 3:**
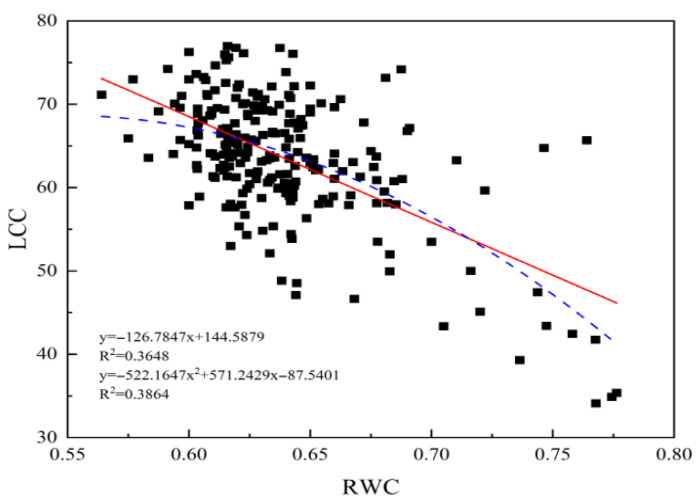
Correlation analysis between LCC and RWC in citrus leaves.

Using this characteristic of plant spectra to diagnose and monitor plant growth status can achieve high analytical accuracy and further explore the correlation between RWC and chlorophyll content. Therefore, in this study, correlation analysis was conducted on 232 collected citrus samples after the LCC and RWC of the leaves were measured, as shown in Figure 3.

Research has shown that the collected sample data are mainly distributed within the range of LCC values of 50–77 and RWC values of 56–66%, indicating an inverse relationship between higher LCC and lower RWC in citrus leaves. The $R^{2}$ for the linear fitting correlation is 0.3648, and the $R^{2}$ for the second-order polynomial fitting correlation is 0.3864. These findings indicate that there is a negative correlation between the LCC and RWC of citrus leaves based on linear and nonlinear predictive models and that the use of hyperspectral characteristic bands of LCC in citrus leaves can have a positive effect on the inversion accuracy of the RWC in citrus leaves.

### 3.2. Comparative Analysis of Prediction Accuracy of Different Wavelet Kernel Functions

**Figure 4:**
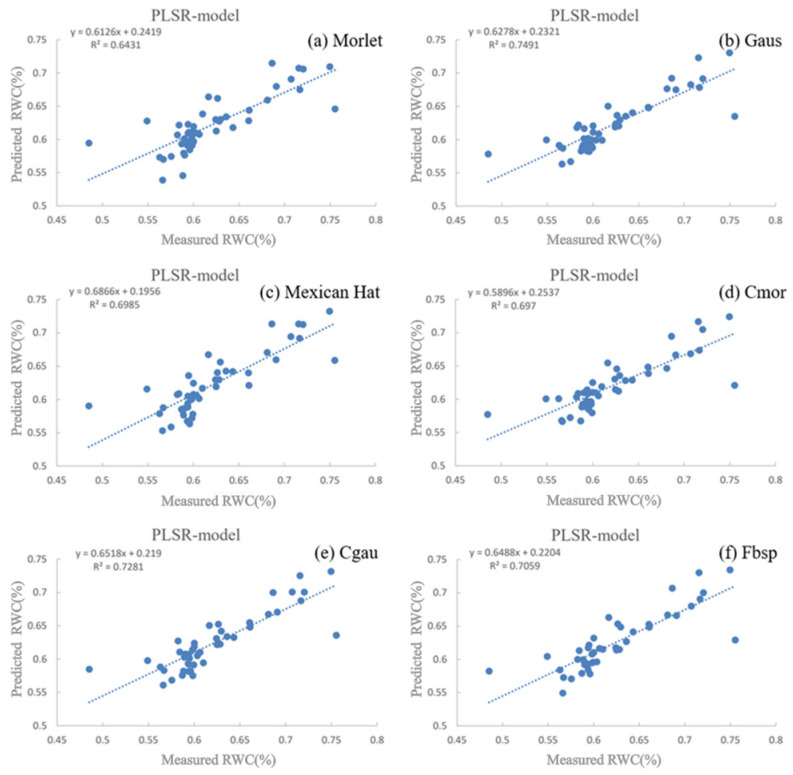
Comparative analysis of optimal prediction models for RWC based on different wavelet kernel functions ((**a**) is Morlet wavelet, (**b**) is Gauss wavelet, (**c**) is Mexican Hat wavelet, (**d**) is Cmor wavelet, (**e**) is Cgau wavelet, and (**f**) is Fbsp wavelet).

This article compares the prediction accuracy between different wavelet kernel functions by adding them. Unlike Fourier transform, the results of the wavelet transform vary depending on the support length, symmetry, vanishing moment, regularity, and similarity of the wavelet kernel function. As shown in Figure 4, Figure 4a–f show the prediction model for predicting citrus leaf moisture content by decomposing each wavelet function into 10 scales and using SPA to screen feature bands.

Through the comparison of prediction accuracy in Figure 4, it is found that the inversion accuracy of Gaus1 wavelet is better than other wavelet functions. Compared with other wavelets, Gaus1 wavelet does not exhibit significant oscillations or abrupt changes, making signal processing and analysis more stable. It can effectively handle noise and non-stationary signals and has good applications in agricultural information monitoring. Therefore, the Gaus1 wavelet has the best effect in inverting the moisture content of citrus leaves.

### 3.3. Comparative Analysis of Citrus Leaf RWC and LCC Prediction Models Based on CWT

**Figure 5:**
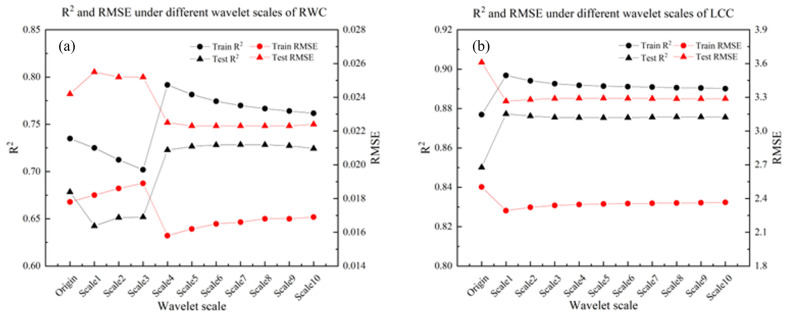
PLSR inverse model results. (**a**) Corresponding performance indicators of RWC predictive model; (**b**) corresponding performance indicators of LCC predictive model (black for $R^{2}$ and red for RMSE; circular symbols for training set data and triangular symbols for testing set data).

This study used CWT to perform multi-scale decomposition on the raw spectral data of 232 citrus leaf samples, generating a total of 10 feature scales (as Scale1 to Scale10). Based on the decomposed features at various scales, quantitative predictive models for RWC and LCC were constructed using the PLSR method. The model performance was evaluated using $R^{2}$ and RMSE, and the results are shown in Figure 5. Among them, Figure 5a shows the trend of accuracy indicators of RWC predictive models at different wavelet scales, while Figure 5b presents the corresponding performance indicator distribution of LCC predictive models. This analysis aims to reveal the differential impact of CWT multi-scale decomposition on the inversion accuracy of two physiological parameters.

Figure 5 shows that after CWT decomposition, the prediction accuracy of Scale4-10 in the RWC training set shows a decreasing trend, whereas the prediction accuracy of Scale1-10 in the LCC training set shows a decreasing trend. The main reason for this phenomenon is that during the CWT decomposition process, the effective information and high-frequency noise of key biochemical parameters such as RWC and LCC in citrus leaves will be redistributed at different scales. Due to the overlap between some effective signals and noise in the frequency domain, certain wavelet scales may contain both useful information and noise components, thereby reducing the prediction accuracy of the model. Although some wavelet scales experience a slight decrease in inversion accuracy due to noise interference, overall, multiple scales decomposed by CWT significantly improve the prediction accuracy of RWC and LCC in citrus leaves. Compared to the original spectral data, CWT can effectively separate noise and enhance key spectral features, resulting in an increase in model $R^{2}$ and a decrease in RMSE at most scales, thus verifying the advantages of CWT in improving spectral inversion performance. The $R^{2}$ of the original RWC data is 0.6784, and the optimal scale of the RWC predictive model is Scale7 ($R^{2}$ = 0.7285). The $R^{2}$ of the original LCC data is 0.8501, and the prediction accuracy of the original LCC data is improved to some extent. The optimal scale for the C predictive model is Scale1 ($R^{2}$ = 0.8773), indicating that the CWT can improve the inversion accuracy of relevant indicators to some extent [26,27].

### 3.4. Sensitivity Band Analysis of Citrus Leaf Moisture Content and Chlorophyll Based on Feature Optimization

On the basis of CWT decomposition, this study further used SPA to screen feature bands highly correlated with RWC and LCC in citrus leaves. This method extracts informative and low redundancy key features from multi-scale wavelet coefficients by minimizing variable collinearity, effectively optimizing the input variable set of the model. The characteristic bands selected by SPA ensure a high leaf water content and chlorophyll content $R^{2}$ while maintaining the RMSE within the error range.

**Figure 6:**
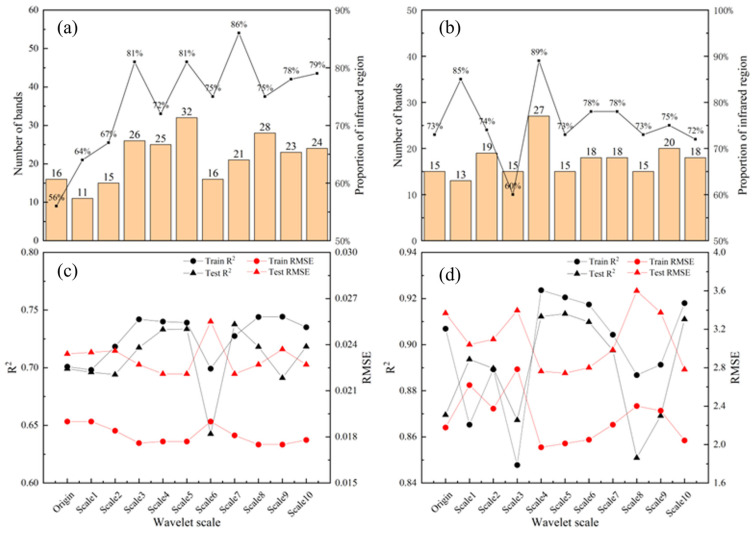
Sensitive band analysis and CWT-SPA-PLSR inversion results. (**a**) Sensitive band analysis of RWC; (**b**) sensitivity band analysis of LCC; (**c**) corresponding performance indicators of RWC predictive model; and (**d**) corresponding performance indicators of LCC predictive model (black for $R^{2}$ and red for RMSE; circular symbols for training set data and triangular symbols for testing set data).

Figure 6a,b show the sensitive band screening results of RWC and LCC in citrus leaves at different wavelet scales, respectively. Among them, Figure 6a shows the number of RWC related feature bands selected by SPA at various scales and their proportion in the total sensitive bands, while Figure 6b presents the selection of feature bands corresponding to LCC. The two figures reflect the differences in the ability of different wavelet decomposition scales to extract moisture and chlorophyll-sensitive information. The distribution characteristics of sensitive bands selected by SPA for RWC and LCC are similar, with most of the characteristic bands distributed in the near-infrared and shortwave-infrared bands. The RWC achieved the highest proportion of 86% at Scale7, whereas the LCC achieved the highest proportion of 89% at Scale 4. The research results indicate that spectral features in the near-infrared (NIR) and shortwave-infrared (SWIR) bands have stronger sensitivity for the inversion of RWC and LCC in citrus leaves. Figure 6c shows the $R^{2}$ and RMSE of RWC at different wavelet scales, and Figure 6d shows the $R^{2}$ and RMSE of LCC at different wavelet scales. Combining Figure 6a,b shows that while reducing the number of bands used to invert the raw RWC and LCC data and each wavelet scale, the prediction accuracy of each predictive model can also be improved. This finding indicates that SPA can improve the inversion accuracy of regression predictive models by reducing redundant data in raw spectral data and selecting feature band combinations with high correlations with RWC and LCC indicators from the entire band.

Figure 6c,d show that when the PLSR predictive model is constructed, the $R^{2}$ of most wavelet scales of citrus leaf RWC and LCC are improved compared with the $R^{2}$ of the original data, while the RMSE is also reduced. Among them, RWC has the best inversion effect when SPA is used to screen 21 feature bands at Scale7 ($R^{2}$ = 0.7376), and LCC has the best inversion effect when SPA is used to screen 15 feature bands at Scale 5 ($R^{2}$ = 0.9134), without overfitting or underfitting. These findings indicate that SPA screening feature bands based on CWT can more effectively improve the PLSR of citrus leaf RWC and LCC.

### 3.5. Analysis of RWC Inversion Results of Citrus Leaves Using RWC–LCC-Sensitive Band Combination

**Figure 7:**
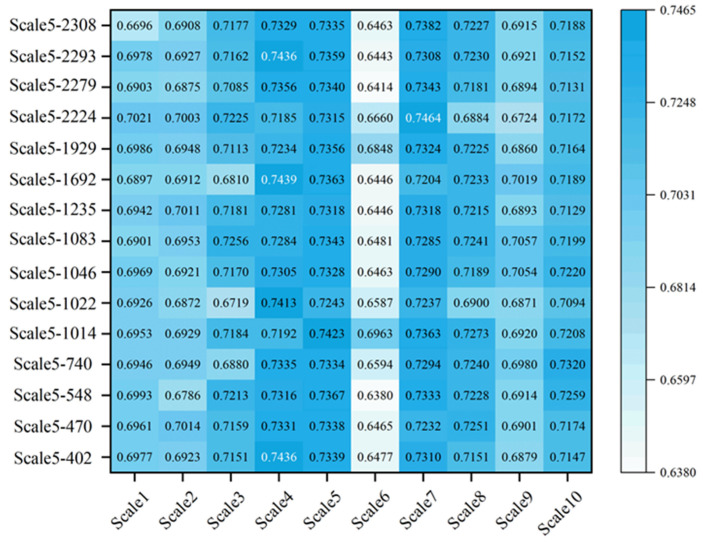
The $R^{2}$ test set for the sensitive band of the joint LCC optimal predictive model with different wavelet scales in RWC.

To optimize the performance of the model, this study uses SPA filtering strategy to extract the most sensitive bands that are most correlated with leaf RWC and LCC from 10 wavelet scales. The PLSR model is constructed by integrating the sensitive bands selected by SPA at 10 scales of RWC, and then the model is established by combining the feature bands corresponding to the scale with the best LCC inversion accuracy. As shown in Figure 7, after the LCC-sensitive bands were combined, not all sensitive bands could reflect RWC well, and the combination of different wavelet scales of RWC did not improve the accuracy. The $R^{2}$ values of the test set are all above 0.7. Among them, the combination of the RWC Scale7 and LCC Scale5-2224 nm bands achieved the highest accuracy ($R^{2}$ = 0.7464), which is in the shortwave-infrared range. This finding indicates that adding characteristic bands within the sensitive band range can improve the model accuracy. The research results indicate that the sensitive bands selected by the LCC optimal predictive model are equally effective in improving the prediction accuracy of leaf RWC. This discovery not only validates the synergistic response mechanism between LCC and RWC spectral features, but also reveals the feasibility of indirectly optimizing water content inversion through chlorophyll-sensitive bands, providing new ideas for inversion of citrus leaves.

**Figure 8:**
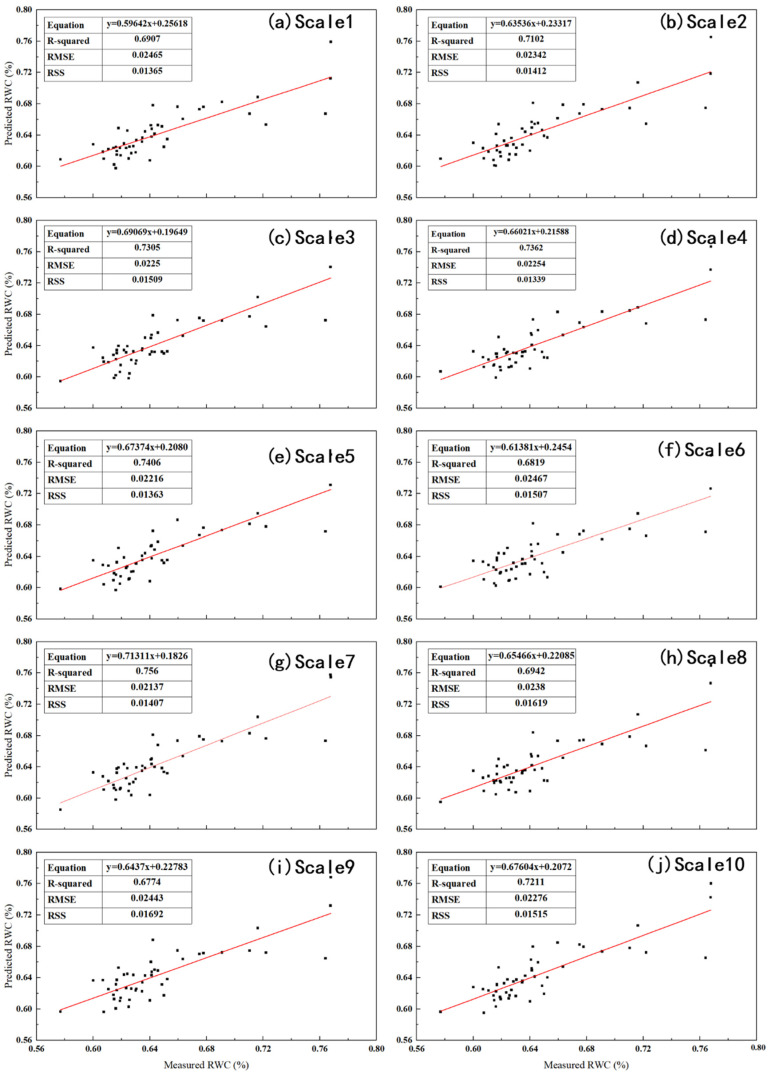
Regression curve of Scale1-10 wavelet scale prediction model.

To explore whether the PLSR model combined with more sensitive bands of the LCC optimal predictive model can better perform RWC, based on the combination of the LCC single sensitive band, the characteristic bands of LCC distributed in the sensitive bands of the near-infrared and shortwave-infrared bands and the wavelet scales of RWC were tested in more bands. Finally, the combination of Scale5-2224 and Scale5-2308 of RWC and LCC on the SWIR can further improve the inversion accuracy of the model, as shown in Figure 8. Figure 8 shows the PLSR curve constructed by the combination of RWC wavelet scales and Scale5-2224 and Scale5-2308. The table contains the regression equation, $R^{2}$, RMSE and sum of squares of residuals (RSS). RSS represents the sum of squared differences between model predictions and actual observations, reflecting the random error components that cannot be described by explanatory variables during the prediction process. The smaller the RSS is, the better the fitting degree. Figure 8 shows that the RMSE and RSS of each wavelet scale of RWC are relatively small, which indicates that the accuracy, stability, and fitting degree of the model are good, and the inversion effect of the Scale7 combination is the best. $R^{2}$, RMSE, RSS, and RPD are 0.756, 0.02137, 0.01407, and 2.02, respectively, which shows that the sensitive band of the best LCC predictive model of more citrus leaves can better reflect the RWC of citrus leaves and improve the inversion accuracy of the RWC of citrus leaves to a certain extent.

## 4. Discussion

The chlorophyll content, as a core indicator of a plant photosynthetic system, plays a dual regulatory role in the carbon water cycle of terrestrial ecosystems [28]. The measured data of this study revealed a significant negative correlation between RWC and LCC in citrus leaves, which was consistent with existing research results [29]. Given the synergistic mechanism of leaf RWC and LCC in plant physiological processes, the academic community has increasingly focused on their coupling research in recent years. This research paradigm is mainly reflected in three dimensions: functional relevance, technical methodology, and application value. The high-precision monitoring data of RWC and LCC provide key indicators for analyzing the coupling mechanism between plant water and nutrients [30]. Coupling chlorophyll fluorescence parameters can improve the sensitivity of a model to water content indicators [31]. The variation characteristics of water and chlorophyll content can reflect the physiological status of crops to provide spatiotemporal change information [32]. This study confirms that there is a significant spectral feature coupling relationship between RWC and LCC in citrus leaves. The LCC-sensitive band can be used to optimize the inversion of RWC index and improve the accuracy of the citrus leaf RWC predictive model.

Studies have shown that spectral responses vary significantly under different water availability conditions [33]. Water absorption strongly influences radiation in the visible and near-infrared ranges [34], with crop water status altering spectral reflectance patterns. This study reveals that the unique spectral characteristics of water molecules play a decisive role in shaping the NIR reflectance spectra of plants. To address the redundancy among adjacent spectral bands, this study employed the Successive Projections Algorithm to identify key feature wavelengths. Spectral sensitivity analysis showed that the spectral response of RWC in citrus leaves exhibited significant band specificity, with greater sensitivity in NIR and SWIR. This finding aligns with previous research, which has consistently identified NIR and SWIR bands as critical for estimating leaf water content across various plant species. For instance, studies on winter wheat demonstrated that Competitive Adaptive Reweighted Sampling selected sensitive bands predominantly located in within the NIR and SWIR ranges [30]. Similar patterns have been observed in grapevines, dragon blood trees, and other species [35]. While some research indicates that visible-region bands contribute less to RWC estimation, most evidence supports the dominance of NIR and SWIR wavelengths. Given the success of sensitive band-based approaches in crop water content assessment, leveraging these key spectral regions for citrus leaves can enhance the accuracy of RWC predictive models. This approach minimizes data redundancy while maximizing predictive performance.

To enhance the estimation accuracy of citrus leaf water content, this study employed CWT to denoise hyperspectral data, remove background interference, and resolve overlapping spectral features. The CWT-decomposed spectra were analyzed alongside key wavelengths selected by the SPA to identify the most sensitive bands for RWC prediction. The results demonstrated that combining LCC-sensitive bands with wavelet coefficients from Scale5 and Scale7 yielded the highest estimation accuracy. This is attributed to their ability to preserve critical spectral details, including peak and trough positions, while effectively suppressing noise, which is consistent with existing research results [36]. However, as the decomposition scale increased, predictive performance declined due to the redistribution of useful spectral information and amplification of high-frequency noise, which is the same as the existing research results [11]. The superior performance of CWT-processed spectra in RWC estimation can be linked to its strong localization properties [37], which enhance noise reduction and feature extraction. These findings further confirm that CWT effectively refines spectral data, improving model accuracy for water content assessment in citrus leaves.

In terms of cost, it is currently difficult to obtain hyperspectral data from drones. In the future, it is necessary to combine the collection time of citrus leaf moisture content, different citrus varieties, and different growth stages to improve the prediction of citrus leaf moisture content.

## 5. Conclusions

Based on CWT preprocessing, this study used SPA to screen the sensitive bands of RWC and LCC in citrus leaves and then optimized the RWC predictive model by combining the optimal sensitive bands of RWC and LCC to find the best predictive model for estimating RWC in citrus leaves. The final conclusions are as follows:

(1) CWT preprocessing markedly boosts the estimation accuracy of RWC and LCC relative to the **maximum** spectrum (max improvements: 6% and 3%), proving it enhances spectral sensitivity to these two indices in citrus leaves;

(2) Combining CWT and SPA, the resulting predictive model showed higher inversion accuracy than the original spectra;

(3) Integrating RWC Scale7 and LCC Scale5-2224/2308 features, the CWT-SPA fusion model showed optimal predictive performance (R^2^ = 0.756, RMSE = 0.0214), confirming the value of multi-scale feature joint modeling. Overall, CWT-SPA coupled with LCC spectral traits can boost the spectral response signal of citrus leaf RWC, enhancing its prediction capability and stability.

## Data Availability

The original contributions presented in this study are included in the article. Further inquiries can be directed to the corresponding author.
